# Physiological Response of Quality Cardiopulmonary Resuscitation, Crossover Trial on Mannequin in Extreme Temperature Conditions

**DOI:** 10.3390/ijerph17165835

**Published:** 2020-08-12

**Authors:** José Luis Martin-Conty, Begoña Polonio-López, Clara Maestre-Miquel, Alicia Mohedano-Moriano, Carlos Durantez-Fernández, Laura Mordillo-Mateos, Jesús Jurado-Palomo, Antonio Viñuela, Juan José Bernal-Jiménez, Francisco Martin-Rodríguez

**Affiliations:** 1 Faculty of Health Sciences, Universidad de Castilla la Mancha, 45600 Talavera de la Reina (Toledo), Spain; JoseLuis.MartinConty@uclm.es (J.L.M.-C.); Begona.Polonio@uclm.es (B.P.-L.); Clara.Maestre@uclm.es (C.M.-M.); Alicia.Mohedano@uclm.es (A.M.-M.); Carlos.Durantez@uclm.es (C.D.-F.); Laura.Mordillo@uclm.es (L.M.-M.); PROFESOR.JJurado@uclm.es (J.J.-P.); PROFESOR.JJBernal@uclm.es (J.J.B.-J.); 2 Advanced Clinical Simulation Center, School of Medicine, Universidad de Valladolid, 47005 Valladolid, Spain; fmartin@saludcastillayleon.es

**Keywords:** hostile thermal environment, physiological fatigue, quality CPR, simulation

## Abstract

Background: To determine the relationship between physiological fatigue and the quality of cardiopulmonary resuscitation (CPR) in trained resuscitators in hostile thermal environments (extreme cold and heat) simulating the different conditions found in an out-of-hospital cardiorespiratory arrest. Methods: Prospective observational study involving 60 students of the health sciences with training in resuscitation, who simulated CPR on a mannequin for 10 min in different thermal environments: thermo-neutral environment (21 °C and 60% humidity), heat environment (41 °C and 98% humidity) and cold environment (−35 °C and 80% humidity). Physiological parameters (heart rate and lactic acid) and CPR quality were monitored. Results: We detected a significant increase in the number of compressions per minute in the “heat environment” group after three minutes and in the mean rate after one minute. We observed a negative correlation between the total number of compressions and mean rate with respect to mean depth. The fraction of compressions (proportion of time in which chest compressions are carried out) was significant over time and the mean rate was higher in the “heat environment”. Physiological parameters revealed no differences in heart rate depending on the resuscitation scenario; however, there was a greater and faster increase in lactate in the “heat environment” (significant at minute 3). The total proportion of participants reaching metabolic fatigue was also higher in the “heat environment”. Conclusions: A warm climate modifies metabolic parameters, reducing the quality of the CPR maneuver.

## 1. Introduction

According to the European Resuscitation Council (ERC), more than 275,000 people a year suffer an out-of-hospital cardiorespiratory arrest in Europe, with a variable incidence depending on the region, between 38 and 84 cases per 100,000 inhabitants/year [1,2]. According to the Spanish registry for out-of-hospital cardiorespiratory arrest, in more than half of the cases (56.7%), basic life support was carried out before the arrival of the Prehospital Emergency Medical Services (PhEMS) [1]. The immediate performance of cardiopulmonary resuscitation (CPR) protocols become a crucial intervention for the survival of the affected patient [1,3]. Survival rates after out-of-hospital cardiorespiratory arrest range from 5.0% to 30.0% in different European countries, increasing considerably in those cases where early CPR is performed [2,4].

Intervening quickly increases the victim′s chances of survival. Equally important is that the intervention meets CPR quality standards, which the American Heart Association (AHA) published in its 2015 guide on quality CPR, which stipulate a frequency of 100 to 120 compressions per minute at a depth of 5 to 6 cm in the lower half of the sternum [5,6].

Because the average arrival time of the PhEMS in Spain is 12 min (8–19 min) [1], the intervention carried out by first responders will involve high-intensity physical exercise that will directly affect the quality of the CPR, which decreases considerably as time passes [7,8,9]. Performing CPR for periods of at least 10 min also affects numerous physiological parameters of the responder (muscle fatigue, heart rate, pulmonary ventilation), which will be less pronounced if the responder is physically fit [9,10,11,12].

In Spain, 57.5% of cardiorespiratory arrests occur outside the hospital setting, so on many occasions the environmental circumstances in which the initial CPR is carried out will not be controlled [13]. Studies on the quality of CPR maneuvers performed in extreme contexts (resuscitation after rescue from water, in a hypoxic environment, with personal protective equipment) are becoming more frequent [14,15,16,17,18]. For this reason, simulating CPR in highly stressful settings could contribute to alleviate situational anxiety and improve the quality of out-of-hospital interventions in these settings [19].

The objective of this study was to determine the relationship between physiological fatigue and CPR quality in resuscitators with basic CPR training in hostile thermal environments (cold and extreme heat compared to a thermo-neutral environment).

## 2. Materials and Methods

### 2.1. Study Design

We conducted a prospective observational cohort study including volunteers over the age of 18 years with accredited basic knowledge of basic life support, all of them students in a Health Sciences degree. The study was carried out at the Faculty of Health Sciences of the University of Castilla la Mancha, in Talavera de la Reina (Toledo, Spain), between 6 and 10 May 2019.

The sample size was calculated with an alpha risk of 0.05 and beta risk of 0.2 with bilateral contrast, assuming three groups of 18 participants each (56 participants), to detect a minimum difference of 0.1 between two groups, and assuming a standard deviation of 0.1 (extracted from previous studies) [14,15]. We estimated a loss to follow-up rate of 5%; hence, the final sample size was 64 participants.

### 2.2. Population

The study was carried out on students from the University of Castilla-La Mancha who participated voluntarily. We included participants between 18 and 65 years, with basic knowledge of CPR (by accredited course or equivalent to training by the AHA or ERC), who read and signed informed consent. Exclusion criteria were: baseline heart rate greater than 120 beats per minute (bpm) or less than 35 bpm; systolic or diastolic blood pressure greater than 160 or 95 mmHg or systolic blood pressure less than 80 mmHg; capillary blood glucose below 65 mg/dL; severe visual or auditory deficiency or some type of functional impairment preventing them from performing the maneuvers; major surgery in the previous 30 days; epilepsy; diagnosed current infections; electrocardiogram with alterations (arrhythmias or changes in the ST segment); oxygen saturation below 92%; body mass index above 40 kg/m^2^; body temperature >38 °C; cutaneous diseases in acute phase or systemic immunological diseases.

**Figure 1:**
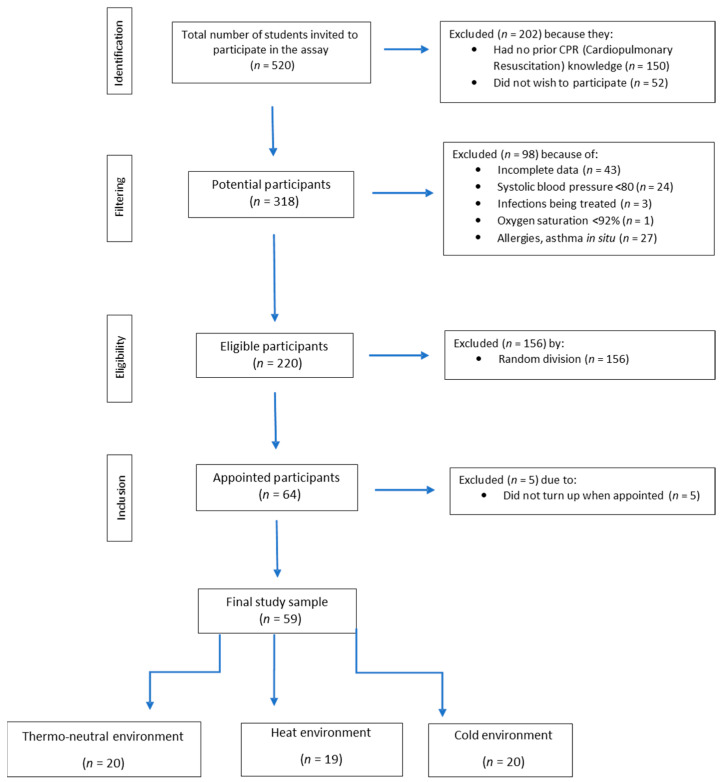
Flow chart of the selection of participants for this study.

Applying the selection criteria and arranging the participants randomly in the different experimental groups, we obtained three homogeneous populations classified as follows: 20 participants in the thermo-neutral environment (eight men and twelve women), 19 participants in the heat environment group (nine men and ten women) and 20 participants in the cold environment group (ten men and ten women) (Figure 1).

In total, 520 students were contacted, 202 of whom were excluded due to not having basic knowledge of CPR or not wishing to participate in the study. Of the remaining 318, we excluded 98 for meeting the exclusion criteria, leaving 220 participants. We randomly selected 64 students in a raffle, of whom five did not turn up to the study, so the final sample was 59 participants (Figure 1).

### 2.3. Study Protocol

We quantified physiological variables lactic acid (LA) and heart rate and analyzed them in relation to the Anaerobic Threshold (AT), which corresponded to LA levels in blood above 4 mmol/L and a calculated maximum heart rate using the Tanaka formula [20]. To assess CPR quality, we recorded mean and optimal depth of the compressions, mean rate, compressions in the optimal frequency zone, compression fraction (proportion of time in which the thoracic compressions are carried out), compression in the optimal zone of depth (5–6 cm) and frequency (100–120 compressions per minute) in a Little Anne QCPR mannequin (Laerdal, Stavanger, Norway).

LA values were obtained with an Accutrend Plus lactometer (Roche Diagnostics, Mannheim, Germany), with a measurement range of 0.8–21.7 mmol/L. The protocol for determining capillary LA consists of the following phases: (1) after turning on the lactometer, test strip code and code on device screen are compared to verify match, and expiration date of reagents is confirmed; (2) blood is drawn from the right index finger using the Solofix^®^ Safety lancet (B. Braun, Melsungen, Germany); (3) 15–40 μL of capillary blood is deposited on the strip and a result is obtained within 60 s; (4) the test strip is removed and the device is cleaned. Heart rate and all quality CPR data were obtained with the X Series^®^ monitor/defibrillator (Zoll, Chelmsford, MA, USA) and the Real CPR Help^®^ CPR system (Zoll, Chelmsford, MA, USA) that provides simultaneous real-time feedback on the depth and frequency of CPR.

The proposed clinical case was identical in all scenarios. The responder had to perform CPR individually for 10 min in a 20 m^2^ laboratory, with a sequence of 30 compressions and two ventilations. The CPR was performed on the floor, positioning the rescuer on the right side of the mannequin. The temperature and humidity of the room were controlled and differed between scenarios: the thermo-neutral environment group performed basic CPR at a temperature of 21 °C and humidity of 60%, the heat environment group at 41 °C and 98% humidity, and the cold environment group at −35 °C and 80% humidity.

Throughout the CPR simulation, heart rate and electrocardiographic rhythm of each volunteer were continuously monitored to assess their constants and immediately detect possible complications. In addition, at minutes 3, 6 and 9, a serial lactic acid curve was established using capillary extractions according to the protocol, while participants minimized CPR during the ventilation phase for sampling. These parameters were measured again 10 min after the end of the simulation.

Clothing and protective equipment used in each scenario were the standard used by the Emergency Services of Castilla-La Mancha.

A randomization sequence was generated using random numbers, according to the gender stratification created with the XLSTAT^®^ BioMED software for Microsoft Excel^®^ (version 14.4.0.) (Addinsoft Inc., New York, NY, USA), so that each subject had the same probability of being allocated to any of the three groups (thermo-neutral environment and two intervention groups). This allocation was coded for the researcher in charge of analyzing the data, and the participants were not informed of it until the beginning of the simulation.

### 2.4. Data Analysis

Descriptive statistics (means ± standard deviation (SD)) were used for quantitative variables and descriptive analysis of frequencies for qualitative variables. The female/male ratio was evaluated using the χ^2^ test. The quantitative variables considered in this study were number of compressions per minute, mean depth, compressions with optimal depth, mean rate, compressions in the optimal frequency zone, compressions in the optimal zone of depth and frequency, as well as the compression fraction. We selected heart rate and lactic acid as additional quantitative variables related to the physiological parameters of the participants.

The physiological baseline of the different variables was established at rest to rule out initial differences between the different experimental groups. We considered the first minute recorded in the experimental protocol as baseline.

We verified that the data met the normal distribution using the Kolmogorov–Smirnov test and then analyzed the data using the ANOVA test. To check if there were significant differences in the variables at the ten time points evaluated (minutes 1–10), we performed a repeated measures ANOVA (intra-subject factor: time; inter-subject factor: ambient temperature) with a Bonferroni post-hoc analysis for multiple comparisons between the ten time points.

Because the variables of the physiological parameters (heart rate and lactic acid) were not normally distributed, the Kruskal–Wallis non-parametric test was used at the five evaluated time points, i.e., baseline, minute 3, minute 6, minute 9 and ten minutes after concluding the simulation. As a post-hoc analysis we applied the Mann–Whitney test between the different scenarios.

Pearson′s χ^2^ test was used to analyze the appearance of fatigue in those participants who exceeded the theoretical anaerobic threshold (TAT) according to the Tanaka formula [20] and lactic acid levels in blood above 4 mmol/L.

The Pearson′s correlation coefficient (ρ) was used to determine the degree of association between the variables’ total compressions in ten minutes and the mean rate with respect to the variable mean depth.

We used SPSS version 24 (SPSS Inc., Chicago, IL, USA).

Ethical approval for this study was granted (22 January 2019) by the clinical research ethics committee of Talavera de la Reina, Toledo (CEIC178013/113).

Reporting of the study conforms to the STROBE (Strengthening the Reporting of Observational Studies in Epidemiology) statement along with references to STROBE statement and the broader EQUATOR (Enhancing the Quality and Transparency of Health Research) guidelines [21].

## 3. Results

No significant differences were observed in the mean age of the participants or the female/male ratio between the three experimental groups (*p* > 0.05) (Table 1).

The variables studied at baseline did not show significant differences between the three experimental groups (*p* > 0.05).

**Figure 2:**
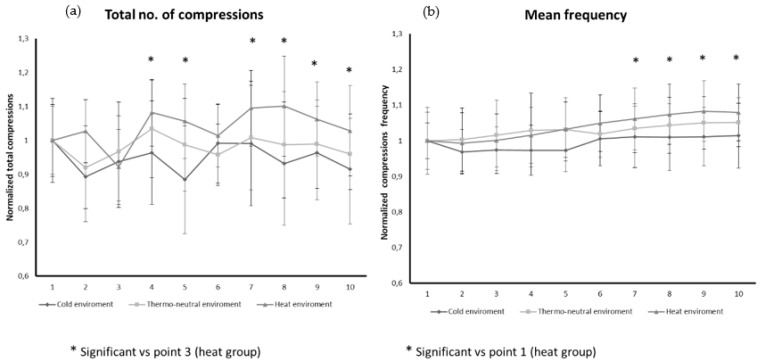
(**a**) Total numbers of compressions per minute according to the studied subgroups. * Significant vs. point 3. (**b**) Mean frequency per minute according to the studied subgroups. * Significant vs. point 1. Data are normalized to baseline values for each group.

When we analyzed if there were significant differences in relation to ambient temperature, we identified a significant effect over time in the variables’ total compressions (*p* < 0.001) and mean frequency (*p* = 0.047) through the repeated measures ANOVA with Bonferroni. Post-hoc analysis showed significant effects in the “heat environment” group. A significant increase in the number of compressions per minute was observed at minutes 4, 5, 7, 8 and 9 compared to minute 3, which showed a decrease in the number of compressions per minute (Bonferroni M4 vs. M3: *p* < 0.001; M5 vs. M3: *p* = 0.017; M6 vs. M3: *p* > 0.05; M7 vs. M3: *p* < 0.001; M8 vs. M3: *p* < 0.001; M9 vs. M3: *p* = 0.007; M10 vs. M3: *p* > 0.05) (Figure 2).

Regarding the mean rate, a significant increase was observed from minute 7 to 10 with respect to minute 1. (Bonferroni M7 vs. M1: *p* = 0.003; M8 vs. M1: *p* < 0.001; M9 vs. M1: *p* < 0.001; M10 vs. M1: *p* < 0.001) (Figure 2).

We found a negative correlation between the variables’ total compressions and mean depth (Pearson: ρ = −0.509; *p* = 0.026): as the total compressions increased, the developed mean depth decreased. Furthermore, the mean frequency also presented a negative correlation with respect to the mean depth (Pearson: ρ = −0.725; *p* < 0.001).

Analyzing the compression fraction variable, we identified significant effects over time with the repeated measures ANOVA (*p* < 0.001).

**Figure 3:**
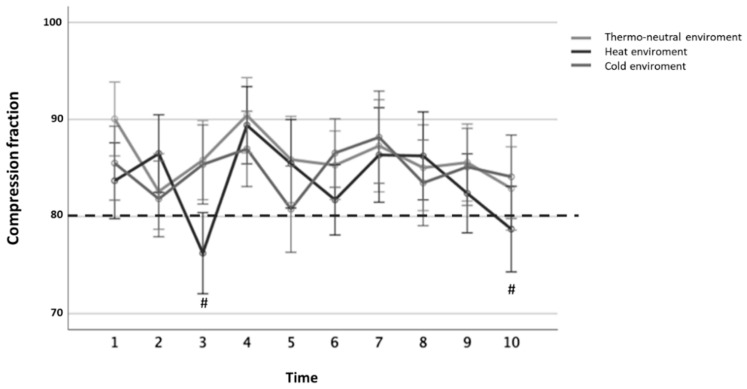
Fraction of correct compressions per minute according to the studied subgroups (percentage ± 2 standard deviations).

Post-hoc tests showed that significantly different values were obtained in the heat environment from minute 2 onwards (*p* < 0.05). However, considering that the compression fraction is considered effective in CPR maneuvers with values greater than 80% (compressions interrupted less than 20% of the total CPR time). Only in the heat environment, values below this limit were recorded in minutes 3 and 10 (Figure 3).

For global values of the different variables studied (total sum of the means obtained during the 10 min), the multifactorial ANOVA showed significant differences between the different experimental groups in mean rate, being higher in the heat environment group (Table 1).

No statistically significant difference was found in changes in the study participants′ heart rate over time between the three experimental groups. A greater and faster increase of lactic acid could be observed in the participants allocated to the heat environment. This increase was significant at minute 3 (*p* < 0.001). Recovery in this group was better after CPR was completed, although these results did not reach statistical significance. The three groups presented higher heart rate and lactic acid results 10 min after concluding the simulation than at baseline, although none of the measurements exceeded the TAT (Table 2).

The heart rate of some participants exceeded the AT at minutes 6 and 9, although without statistically significant differences between groups. No differences between groups were identified in the percentage of participants that exceeded 4 mmol/L of in lactic acid in any of the measurements, although a different pattern was observed in each group. More than 50% of the participants in the heat group exceeded the threshold during CPR (73.7% at minute 6), although they recovered more efficiently after concluding the simulation. In contrast, lactic acid in the thermo-neutral environment group did not increase as much, although their recovery after finishing CPR was poor: the same percentage of participants had high lactic acid 10 min after the simulation as in minute 9 of the intervention. The increase in lactic acid occurred more slowly in the cold environment group, without ever exceeding 50% of the participants above 4 mmol/L (Table 3).

## 4. Discussion

The results of the study suggest an association between CPR quality and adverse climatic conditions in a heat environment with high ambient humidity (41 °C and 98% humidity). However, the CPR quality in a cold environment (−35 °C and 80% humidity) did not differ significantly from that in the thermo-neutral environment (21 °C and 60% humidity).

All participants performed the CPR for 10 min, and the heat environment group suffered a decrease in CPR quality. These data agree with a study by Mora Rodríguez and Aguado Jiménez [22] in which they describe that athletes in warm environments (39 ± 1 °C), not acclimatized to heat, have high levels of lactic acid and a higher heart rate compared to neutral temperature (21 ± 2 °C). Not only does prolonged exercise in heat conditions increase lactic acid production, but it is also a short test of 20 min [22].

In any of the studied environments, the lactic acid levels at the baseline were not significantly different as might be expected. Due to physical exertion, the levels of lactic acid in all cases increased, until reaching their threshold at minute 6. From that point on, serum lactic acid levels dropped in all groups, as it began to be metabolized. Lactic acid produced during physical activity (more than 90%) is recycled and converted to pyruvate [23]. In heat environments, lactic acid values increased or reached the threshold more quickly, although once it had been reached, its behavior was similar to that in the other environments, hence the remaining time points had no statistically significant differences [22]. Furthermore, the participants in a warm environment had a greater recovery effect than those in other environments.

Although the mean of the groups was far from exceeding the TAT, a high percentage of individual participants exceeded this threshold in the measurements at minute 6 (between 30% and 45%) and at minute 9 (between 20% and 30%). These data suggest that participants reached metabolic fatigue.

The data from our study indicate that only in the first 3 min of CPR in a heat environment, are a number of correct compressions achieved. After the third minute, the percentage of correct compressions decreases progressively, as the number of compressions per minute increases and the depth of the compressions decreases, as observed in similar studies [24]. Therefore, a fatigued responder would only be capable of maintaining adequate CPR quality for the first three minutes in a heat environment, unlike in the studies by Ochoa et al. [24] and Ashton et al. [25], who affirmed that the fatigue caused by a CPR maneuver transcended in its quality from the first minute.

In contrast, changes in the ambient temperature modify musculoskeletal aspects [26]. At high temperatures the contribution of the number of motor units of fast-contracting fibers increases, which could explain the greater number of compressions carried out by the participants of the heat group at minutes 4, 5, 7, 8 and 9 with respect to minute 3.

The absence of metabolic adaptation is in line with the gradual decrease in the depth of compressions observed in all the groups, and which, in the case of our study, directly diminishes the quality of the said parameter throughout (relative to the appearance of fatigue in the participants as demonstrated in previous research) [16].

Recently, Barcala-Furelos et al. [27] carried out a similar study analyzing the impact of extreme heat on rescuers during CPR. As opposed to us, they did not find significant differences on the quality of the CPR regarding the control group; although in their case, a period of 50 min of acclimation was included at the end. That could influence on the musculoskeletal adaptation of the participants.

Various confounding factors have been taken into account in our conclusions, especially aspects such as the continuous observation of the researchers while performing the CPR, the visualization of the quality of the CPR on the simulator monitor or the momentary interruption of the test to measure lactate at minutes 3, 6 and 9 of the CPR simulation, which was performed during the ventilation phase to minimize its impact. We also took into account the protective equipment worn by the participants who carried out the simulation in a cold environment, which corresponded to that used by the emergency services in winter. This was done with the aim of minimizing the confounders and to adapt the simulation as well as possible to the real conditions encountered in out-of-hospital resuscitation.

The main strength of this study is that we recruited students of the health sciences with basic knowledge of CPR, which favors the representativeness of the results due to the homogeneity of the sample. This study was strengthened by including scenarios of extreme temperatures of cold and heat that involved a complex use of resources.

The main limitation is that we found no study to compare these findings with in relation to how extreme weather affects the performance of CPR (only the study from Barcala-Furelos et al. [27]), which is why we compared our results with those in physical exercise physiology or on the appearance of fatigue during CPR simulation. The study sample would also have to be expanded to adapt the results more to the conditions of the population. One could even consider using qualified health professionals to check the reproducibility of the study.

## 5. Conclusions

In conclusion, the simulation carried out in hostile thermal environments allowed us to establish that the increase in lactate can physiologically affect the rescuer′s performance, reducing the quality of the CPR maneuver from the third minute onwards in relation to the appearance of fatigue and musculoskeletal adaptation to the weather.

## Figures and Tables

**Table 1:** Differences in parameters in genders and the studied subgroups.

Variable	Thermo-Neutral Environment	Heat Environment	Cold Environment	*p*-Value **
Number (*n* (%))	20 (33.9%)	19 (32.2%)	20 (33.9%)	
Age *	20.85 ± 2.94	22.95 ± 2.65	20.40 ± 2.14	0.08
Gender				0.806
Men (*n* (%))	8 (40%)	9 (47.4%)	10 (50%)	
Women (*n* (%))	12 (60%)	10 (52.6%)	10 (50%)	
Mean depth *	4.97 ± 0.38	5.03 ± 0.42	4.97 ± 0.3	0.874
Optimum depth of compressions *	410 ± 200	445 ± 204	455 ± 182.2	0.740
Mean frequency *	114.5 ± 8.0	116.7 ± 7.91	110.4 ± 7.33	0.047
Compressions in optimum zone *	598.3 ± 239.4	464.1 ± 207.1	598.8 ± 172.8	0.070
Compression fraction *	84.8 ± 6.13	83.7 ± 5.82	86.1 ± 5.78	0.448
Compressions in optimum depth zone and optimum frequency *	289.5 ± 214.7	259.8 ± 165.6	319.2 ± 142.6	0.582

* Data expressed as mean + standard deviation. ** *p*-value of the multifactorial ANOVA.

**Table 2:** Mean and standard deviation of heart rate and lactic acid per studied subgroups.

Variable	Thermo-Neutral Environment	Heat Environment	Cold Environment	*p*-Value **
HEART RATE				
Baseline *	79.4 ± 15.4	78.32 ± 13.0	84.2 ± 14.9	0.467
Min. 3 *	135.6 ± 15.1	134.81 ± 17.2	135.2 ± 16.3	0.979
Min. 6 *	161.1 ± 26.4	153.9 ± 28.3	155.9 ± 22.0	0.737
Min. 9 *	158.9 ± 16.9	155.9 ± 21.8	156.8 ± 21.1	0.709
Rest at 10 min. *	96.1 ± 12.2	91.6 ± 15.3	99.7 ± 16.3	0.339
LACTIC ACID				
Baseline *	2.2 ± 1.5	1.9 ± 1.0	1.9 ± 1.2	0.734
Min. 3 *	3.8 ± 2.0	6.7 ± 5.4	3.0 ± 2.4	<0.001
Min. 6 *	4.9 ± 2.8	6.3 ± 4.2	5.2 ± 4.9	0.356
Min. 9 *	4.1 ± 2.6	5.1 ± 3.3	5.0 ± 4.2	0.494
Rest at 10 min. *	3.5 ± 1.3	2.8 ± 1.5	2.8 ± 2.1	0.097

* Data expressed as mean ± standard deviation. ** *p*-values of the non-parametric Kruskal-Wallis test.

**Table 3:** Number and percentage of subjects who exceeded the theoretical anaerobic threshold and lactic acid of 4 mmol/L per the studied subgroups.

Variable	Thermo-Neutral Environment	Heat Environment	Cold Environment	*p*-Value **
HEART RATE > Anaerobic threshold				
Baseline *	0	0	0	-
Min. 3 *	0	0	0	-
Min. 6 *	9 (45%)	6 (31.6%)	6 (30%)	0.555
Min. 9 *	4 (20%)	5 (26.9%)	6 (30%)	0.764
Rest at 10 min. *	0	0	0	-
LACTIC ACID > 4 mmol/L				
Baseline *	2 (10%)	1 (5.3%)	2 (10%)	0.830
Min. 3 *	8 (40%)	11 (57.9%)	4 (20%)	0.052
Min. 6 *	12 (60%)	14 (73.7%)	9 (45%)	0.189
Min. 9 *	8 (40%)	13 (68.4%)	9 (45%)	0.168
Rest at 10 min. *	8 (40%)	3 (15.8%)	4 (20%)	0.175

* Data expressed as *n* (%). ** *p*-value of Pearson’s χ^2^.
